# Owners’ Attitudes, Knowledge, and Care Practices: Exploring the Implications for Domestic Cat Behavior and Welfare in the Home

**DOI:** 10.3390/ani9110978

**Published:** 2019-11-15

**Authors:** Emma K. Grigg, Lori R. Kogan

**Affiliations:** 1 Department of Population Health and Reproduction, School of Veterinary Medicine, University of California, Davis, CA 95616, USA; 2 Clinical Sciences Department, Colorado State University, Fort Collins, CO 80523, USA; lori.kogan@ColoState.edu

**Keywords:** companion animal, human-animal bond, behavior, welfare, cats, *Felis catus*

## Abstract

**Simple Summary:**

Available research on the link between domestic cats’ environment and welfare has primarily been conducted on cats living in animal shelters or research facilities; a better understanding of the welfare of cats living in homes is needed. We used an anonymous online survey to investigate the attitudes of current U.S.-based cat owners towards cats as pets; owner knowledge about normal cat behavior and environmental needs; current trends in cat care; behavior problems reported for these cats; and the human-animal bond. We found that owners with a more accurate understanding of cat behavior, and stronger reported bond with their cats, reported fewer behavior problems. Conversely, owners’ agreement with certain misconceptions about cats, and high perceived costs of care, were correlated with the use of punishment (e.g., yelling, hitting, or spraying with water) in response to misbehavior. Our results suggest that many cats living in private homes may be receiving only minimal environmental enrichment, particularly in the case of interactive (e.g., playing with a friendly human, or exploring changing environments) vs. static (e.g., cat toys such as stuffed mice) enrichment. Collectively, these results support the premise that better education of cat owners could benefit the welfare of cats living in private homes.

**Abstract:**

Available research on the link between domestic cats’ environment and welfare has primarily been conducted in animal shelters or research facilities; a better understanding of the welfare of cats living in homes is needed. This study measured the attitudes of current U.S.-based cat owners towards cats as pets; owner knowledge about normal cat behavior and environmental needs; current trends in cat care; cats’ behavior in the home; and the human-animal bond. The primary hypothesis was that owners with a more accurate understanding of cat behavior and a stronger reported bond with their cats would report fewer behavior problems. Data from an online, anonymous, cross-sectional survey of 547 cat owners supported the primary hypothesis: owner knowledge, along with two measures of the human-animal bond (owner-pet interactions, and perceptions of affordability of cat ownership), were significant predictors of the number of reported behavior problems. In addition to fewer reported behavior problems, greater owner knowledge about cats was correlated with less use of positive-punishment-based responses to misbehavior, and increased tolerance of potential behavior problems when present. Owners’ agreement with certain misconceptions about cats and perception of high costs of care were correlated with the use of positive punishment in response to misbehavior. Based on the survey results, many cats living in private homes may be receiving only minimal environmental enrichment. Collectively, these results suggest the need for better education of cat owners. Topics could include: understanding normal cat behavior and correcting misconceptions; enrichment needs (particularly of indoor-only cats) and the risk of behavior problems when cats’ needs are not met; welfare risks associated with declawing; and the importance of sufficient resources to minimize social and territorial conflict.

## 1. Introduction

Much of the research on the links between domestic cats’ environment and their welfare has been conducted in animal shelters or research catteries (e.g., influence of environmental stressors and handling on cat health and welfare) [1,2,3]. In recent years, a number of authors have noted the need for a better understanding of the welfare of privately-owned domestic cats (*Felis catus*) living in homes, and have expressed concern that needs of these cats are not being met [4,5,6,7,8]. Several studies have suggested that many cat owners lack rudimentary knowledge necessary for optimal cat care, such as how to prevent unwanted litters or provide basic health care, or how to provide an ideal environment for this species [7,9,10,11,12]. This situation may be particularly serious for cats’ behavioral needs [6,13] with many owners lacking the ability to accurately read their cats’ body language [14]. Yet, at the same time, increasing urbanization has altered many cats’ environments. It has contributed to smaller living spaces, higher population densities, complete indoor confinement, and more time spent alone [15], all of which may contribute to increased stress for cats in the home (either due to insufficient resource availability in multi-cat homes, or social isolation from familiar humans or other compatible cats) [13]. Suboptimal handling, management, and environments increase the risk of behavior problems such as aggression and housesoiling in companion animals (e.g., references [4,6,16,17]). Behavior problems (or species-typical behaviors perceived as problems) can have a negative effect on the emotional bond between companion animals and human caretakers [18] which may in turn impact the care and welfare of the cat [4,8,13], creating a negative self-perpetuating cycle by increasing the frequency or severity of behavior problems. The outcome of this cycle for the cats involved is often dire; behavior problems are one of the most common reasons for relinquishment and/or euthanasia of pet cats [19,20,21].

Taken collectively, these issues point to the necessity of conducting a needs assessment [22] to better understand cat owners’ attitudes and practices, with the goals of identifying detrimental gaps in owner knowledge and skills, and thereby targeting educational campaigns to improve owner understanding of normal cat behavior and species-specific needs. Correcting misconceptions and providing accurate, evidence-based information pertaining to domestic cats’ needs can help improve the human-feline bond, and as result, help reduce risks to cat welfare. Therefore, the purpose of this cross-sectional survey was to measure the attitudes of current U.S.-based cat owners towards cats as pets, their knowledge about normal cat behavior and environmental needs, and current trends in cat care (such as number of cats in the home, prevalence of declawing, provision of outdoor access, and access/availability of resources known or believed to be important to cats). In addition, owners were asked questions related to their cats’ behavior and the strength of their bond with their cats. Our primary hypothesis, based on the current literature, was that owners with a more accurate understanding of cat behavior, and stronger reported bond with their cats, would also report fewer behavior problems. An additional study goal, as noted above, was to identify the gaps in current cat owners’ knowledge and pet care practices, and explore relationships between these factors, as a prelude to the creation of educational campaigns to correct cat owners’ misconceptions and improve their cat-related knowledge.

## 2. Materials and Methods

An online, anonymous, cross-sectional survey was developed using Qualtrics (Qualtrics, Inc., Provo, UT, USA). The survey was designed, reviewed, and tested by the co-investigators and their colleagues (veterinary and applied animal behavior professionals; see author affiliations for institutions) after seeking input from cat-owning representatives from the community. A portion of the survey consisted of questions from published survey measures to assess cat care and owner interactions (such as the Cat Owner Relationship Scale, CORS) [9]. The survey was pilot tested by ten individuals for ambiguity and/or potentially missing or inappropriate response options, with revisions made based on the results of the pilot testing. The final 67-item survey (see Supplementary Materials) and study design were approved by the Colorado State University Institutional Review Board (IRB # 19-9335H). Survey respondents were recruited in August 2019 through Amazon’s Mechanical Turk (MTurk; Amazon Inc., Seattle, WA, USA) platform, an open online marketplace providing affordable access to over 100,000 potential survey respondents [23]. Survey respondents receive monetary compensation for completing surveys, but the amounts received are typically very small (e.g., ≤50 cents per survey), suggesting that survey respondents are internally motivated [23]. Diversity of participants recruited through MTurk is high (more diverse than typical Internet samples or American college-based samples), and the quality of data collected meets or exceeds the psychometric standards considered acceptable in published research in the social sciences [23].

In order to minimize the influence of geographic and cultural differences on respondent data, the survey was made available only to responders residing in the United States. Adult (18 years or older) participants who were the current owners of at least one cat between the ages of 1 and 18 years were recruited for the study, and demographic data were collected (e.g., age group, gender, household information such as presence of children younger than 10 years old, or other cats and dogs in the home). Respondents were asked to state their agreement level using a Likert scale of 1–5 (with 1 indicating ‘strongly disagree’ and 5 ‘strongly agree’) with twelve statements about cats as pets in general (survey items 9–20; see Supplementary Materials). These statements encompass common beliefs and misconceptions about cats (e.g., “Cats can’t be trained to do tricks,” “Cats are naturally aloof and independent”, etc.). Three of these questions had factually right or wrong answers, based on currently available literature (survey items 15, 18, and 19), and responses to these questions were added together to create an “owner knowledge” score for each respondent. Next, they were asked a series of questions about their own cat, including characteristics of the cat (such as age, sex/reproductive status, and whether it is declawed or not); potential behavior problems exhibited by the cat (and the degree to which these behaviors bothered them); and their care of this cat in the home (including resources provided to the cat). If a participant owned more than one cat, they were instructed to choose the cat whose first name started with the letter closest to the first letter of the alphabet, and answer the questions about this cat only. For resources such as litterboxes and feeding stations (food bowls in different locations), the ratio of #items/#cats in household was calculated as an index of each cat’s ability to access these resources.

Because owner reports of ‘misbehavior’ are driven by owner perception of what constitutes a behavior problem (and some behaviors often classified by owners as behavior problems, e.g., housesoiling and vomiting, may in fact be undiagnosed medical-rather than behavioral-issues), owners were asked to report on cat behavior problems in two ways. Survey items 31–39 list specific behaviors and ask owners to report on whether or not they occur (and the degree to which the behavior bothers them); and survey item 49 asks the yes/no question, “Does your cat ever misbehave?” This allowed for comparison of owner responses to the two different question formats. Owners were also asked to report how they respond when their cat misbehaves; response options included varying levels of positive punishment (such as yelling, hitting, spraying with water), ignoring the behavior, or redirecting the cat to a more acceptable behavior. Finally, respondents were asked about their relationship with this cat; utilizing questions from the CORS [9]. As in the CORS, responses were used to calculate three bond-related ‘subscores’ reflecting owner’s interactions with their cat (“interactions”, encompassing mean response to survey items 56, 57, 59, and 60; see Supplementary Materials), their perceived emotional closeness to their cat (“emotional bond”; survey items 62, 63, and 65), and their perceptions of the costs associated with caring for their cat (“affordability”; survey items 58, 61, 64, and 66); higher subscores represented a more positive relationship with their cat. Some responses were reverse coded when combining items for analysis in order to maintain consistency of affect. Due to the broad scope of the present study and the desire to keep the survey to a reasonable length to minimize participant attrition, only select questions from the CORS scale were used. Therefore, validation measures of the CORS may not apply to this study. Descriptive statistics were then calculated to characterize current attitudes and patterns in pet cat management.

In order to explore specific relationships among these data, associations between demographic variables, cat care practices, occurrence of behavior problems, and bond subscores were further investigated using non-parametric approaches best suited to categorical and Likert (ordinal) data [24]: Spearman’s rank order correlations, Mann-Whitney U, Kruskal-Wallis nonparametric analysis of variance and chi-square analyses. This also allowed investigation of variables (e.g., ordinal and categorical data) not suitable for inclusion in the multiple linear regression, described below. To reduce the risk of committing a Type I error when conducting multiple comparisons, significance level (α) for these analyses was set at *p* = 0.01 [10]. Bonferroni corrections were not used, as these would greatly reduce statistical power and markedly increase the risk of committing a Type II error, as noted by Nakagawa [25]. Nonetheless, results of the exploratory bivariate analyses should be viewed with this caveat in mind.

In order to examine the effect of owner attitudes, practices, and household variables on the cats’ behavior, the relationship between a number of these potential predictor variables and the number of behavior problems reported by owners (the response variable) was assessed using multiple linear regression analysis. Results of the exploratory analyses were used to guide selection of potential predictors in the multiple regression model. Potential predictor variables included in the model were: total number of cats living in the home, total number of dogs living in the home, number of children (≤10 years of age) living in the home, owner knowledge score, litterbox ratio (#litterboxes/#cats in home), feeding stations ratio (#separate feeding areas/#cats in home), owner use of positive punishment score, and the three bond-related subscores described above. Significance level (α) for the multiple linear regression was set at *p* = 0.05.

Data were analyzed using SPSS (IBM, Armonk, NY, USA) and XLSTAT (Addinsoft, New York, NY, USA) for Microsoft Excel (Microsoft Corp., Redmond, WA, USA). All tests were two-tailed.

## 3. Results

### 3.1. Characteristics of Owners and Cats Living in Homes (Descriptive Statistics)

#### 3.1.1. Current Patterns of Cat Care and Attitudes towards Pet Cats among U.S. Cat Owners

A total of 547 responses were obtained from the MTurk survey, 39.1% of whom were male, and 60.3% female. Mean age of respondents was 38.0 (±11.5) years; median = 36 years; the mean age of cats reported on in this study was 6.9 (±4.6) years, with approximately 30% of cats aged 2 (19.2%) and 3 (11.5%) years. The majority (93.6%) of respondents had been living with their cat for at least one year (mean duration = 3.2 ± 1.2 years), and most (71.5%) had obtained their cat when the cat was less than one year of age. The majority of cats in the study were spayed females (45.1%) or neutered males (39.7%), with a small proportion intact (8.2% intact males and 7.1% intact females). The majority of cats were not declawed (76.5%), and were kept indoors only (60.0%). The most common acquisition source for cats in this study was a shelter or rescue (41.5% of cats). The mean number of cats in respondents’ homes was 1.8 (±1.4), with 58.0% of respondents living in single-cat homes. Demographic data on survey respondents and their cats are summarized in Table 1.

When asked about resources made available to their cats, most respondents reported providing at least one of the listed resources to their pets (Table 2); the most commonly provided were “quiet, private hiding places” (92.1% of respondents) and “toys for independent play (such as ping pong balls, catnip-stuffed toys, etc.)” (81.3% of respondents). The mean number of litterboxes available per cat was 1.7 ± 0.7 (range: 0.1–4); mean number of feeding stations per cat was 1.0 ± 0.5 (range: 0.1–3). The majority of owners (62.9%) reported scooping the litterbox(es) one or more times per day; the mode for reported cleaning (i.e., scooping out waste) frequency was once per day (45.3% of respondents). The most popular litter type used was clay clumping (61.8%). Nearly half (47.7%) of respondents said they played with their cat (i.e., with an interactive toy like a laser pointer or ‘fishing pole’) at least once per day, and the majority (80.25%) played with their cat at least every few days. Only 3.8% said they never played interactively with their cats. Nearly one third (30.7%) of owners reported having trained their cat (to do tricks, play fetch, use the toilet, etc.). Data on provided resources are summarized in Table 2.

**Figure 1:**
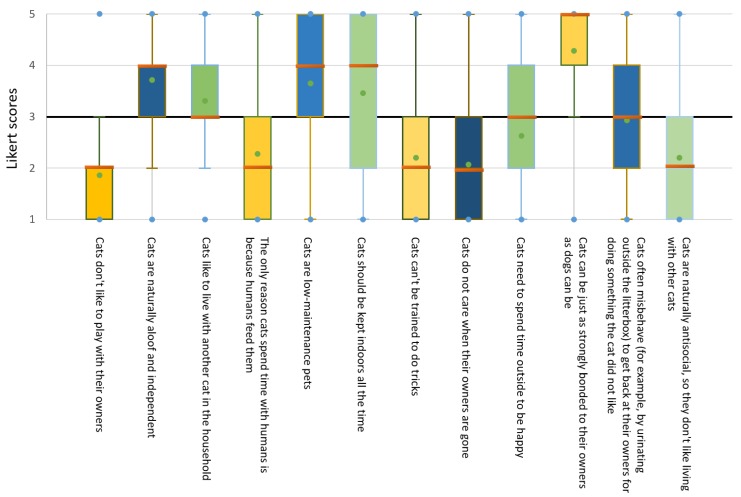
Box plot showing responses by owners to Likert-scale questions about their attitudes to pet cats in general. Response options ranged from 1 (“Strongly Disagree”) to 5 (“Strongly Agree”). Median is indicated by the orange horizontal bar (e.g., at 2 for “Cats don’t like to play with their owners”), upper and lower boundaries represent 1st and 3rd quartiles, and the green dot represents the mean. The midline at Likert score = 3 represents a neutral view, “Neither Agree nor Disagree”; size of box indicates degree of variability among responses, and box position relative to this line indicates where most respondents’ views fell on these questions. Box colors are to aid in discriminating between adjacent boxes only.

Attitudes of current cat owners towards cats as pets in general varied, but reflected primarily positive attitudes towards cats (Figure 1). For example, most owners strongly agreed with the statement that “cats can be just as strongly bonded to their owners as dogs can” (mode = 5, “strongly agree”; 56.7% of respondents), and strongly disagreed with the statement “cats don’t like to play with their owners” (mode = 1, “strongly disagree”; 46.4%). Opinions were more mixed in response to other statements, such as “cats need to spend time outdoors to be happy” (mode = 2; 25.8%) and “cats often misbehave (for example, by urinating outside the litterbox) to get back at their owners for doing something the cat did not like” (mode = 4; 28.9%) (Figure 1).

**Figure 2:**
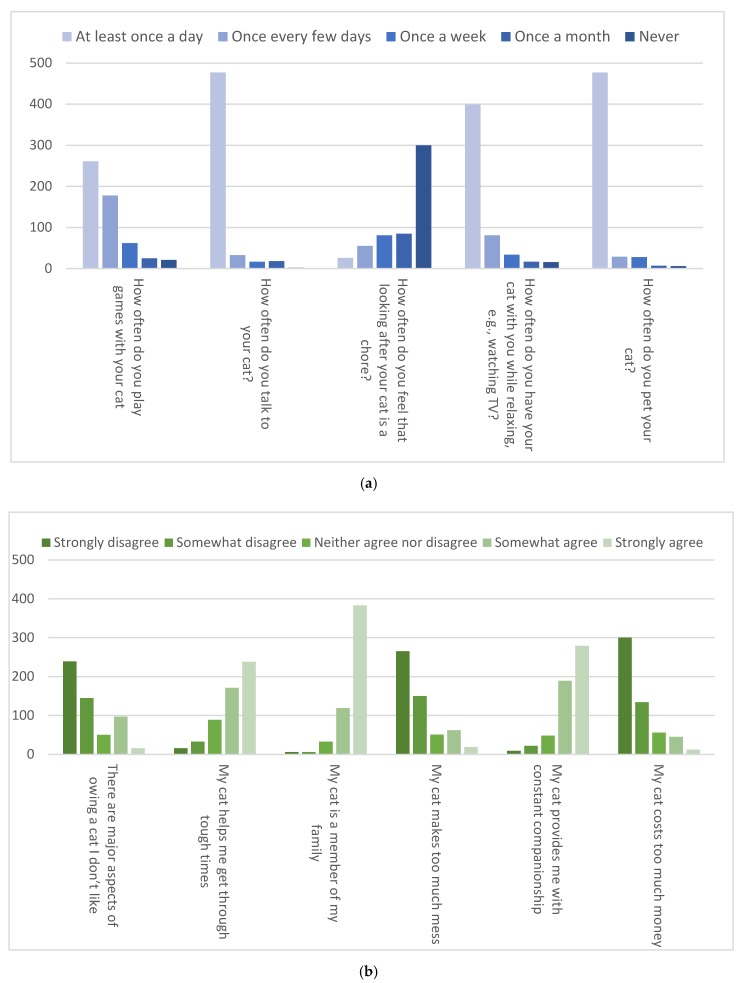
Cat owner responses to questions related to their bond with their cat. (**a**) Response options ranged from 1-“at least once per day” to 5–“never”. (**b**) Response options ranged from. 1–“strongly disagree” to 5–“strongly agree”.

The overwhelming majority (91.8%) of respondents considered their cat as a family member, with 70.0% expressing strong agreement with this statement. The majority of respondents said that their cat provides them with constant companionship (85.6%), and that their cats helps them get through tough times in their lives (74.8%). Most respondents (87.2%) reported talking to their cats at least once per day; the same percentage reported petting their cats at least once per day. Conversely, only about 10.4% of respondents agreed (somewhat or strongly) with the statement “my cat costs too much money”, and 14.8% agreed (somewhat or strongly) with the statement “my cat makes too much mess.” Responses relating to the bond between cat and owner are summarized in Figure 2.

#### 3.1.2. Reported Behavior Problems and Owner Responses

When asked whether their cat had ever misbehaved, 47.0% of respondents said yes, while the remaining 53.0% said no. The most common potential behavior problem reported by owners was “anxiety or fear”, with 59.4% of owners reporting this cat behavior. Note that not all owners were bothered by this behavior (of those respondents reporting this behavior, 48.3% stated that it did not bother them at all). In addition, it should be noted that not all owners who reported this behavior felt it constituted ‘misbehavior’, as evidenced by the lower percentage (47%) of all respondents who answered “yes” to the yes/no misbehavior question [“does your cat ever misbehave?”]. This was also the case for vomiting (54.1% of respondents reported this behavior in their cats). Among the potential behavior problems, aggression towards familiar people had the lowest reported rate, with 24.3% of owners reporting this in their cats (of those reporting this behavior, 37.6% stated that the behavior did not bother them at all). The behaviors that bothered owners the most were housesoiling (with 8.3% of respondents who experienced this behavior reporting that it bothered them a great deal), destructive behavior such as scratching furniture (7.7% reported that it bothered them a great deal), and excessive vocalization, including at night (6.9% reporting that it bothered them a great deal). When queried about their responses to misbehavior, the most commonly used responses included redirecting the cat (90.3% of owners reported using this technique), making a loud noise, such as by handclapping (85.2%), and yelling (77.0%). Approximately half (51.4%) stated that they had used the technique of spraying the cat with water. Only 10.9% of owners reported ever hitting or kicking their cat in response to misbehavior. Fifty respondents (9.1%) said their cat was on some form of medication, but only 12 (2.2%) said their cat was on medication for behavioral reasons (or for both behavioral and physical reasons). The majority of owners (93.2%) said that they had never considered relinquishing their cat because of his/her behavior. Response rates for all surveyed potential behavior problems are shown in Table 3.

### 3.2. Relationships between Owner Demographics, Practices, Measures of the Human-Animal Bond, and Cat Behavior (Exploratory Analyses)

#### 3.2.1. Relationships between Cat Characteristics and Behavior Problems

For all cats, behavior problems were significantly more prevalent in declawed cats (median = 4, range 0–9; *n* = 128) compared to cats that were not declawed (median = 3, range: 0–9; *n* = 417) (U = 21,421, *p* < 0.002). There was no difference in the number of behavior problems reported in male vs. female cats, but there were higher numbers of problems reported for intact (median = 6, range: 0–9, *n* = 81) vs. altered (median = 3, range: 0–9, *n* = 463) cats (U = 12,390.5, *p* < 0.0001). When the sexes were considered separately, there was no significant difference in the number of behavior problems between intact (median = 5, range: 0–9) and neutered (median = 3, range: 0–9) male cats, although the difference approached significance (*p* < 0.02). For females, there were a significantly higher number of behavior problems reported for intact (median = 6, range: 0–9, *n* = 37) than spayed (median = 3, range: 0–9, *n* = 246) cats (U = 2463.4, *p* < 0.0001).

Although the number of behavior problems in cats obtained from pet stores (median = 5, range: 0–9, *n* = 32) was somewhat higher than cats from other sources [e.g., cats found as strays (median = 3, range: 0–9, *n* = 105) or obtained from shelters or rescues (median = 3, range: 0–9, *n* = 225)], the difference was not significant (*p* = 0.045).

#### 3.2.2. Relationships between Owner and Household Characteristics, Including Distribution of Resources, and Behavior Problems

There was a significant direct correlation between respondents’ tendency to use positive punishment and the number of behavior problems reported (r = 0.41, *p* < 0.0001), and a significant but weaker inverse correlation between respondents’ knowledge about cats (as represented by their mean score on the three factual questions) and their use of positive punishment (r = −0.18, *p* < 0.0001) (Table 4). When considering participant responses to the yes/no question “Does your cat ever misbehave?” (survey item 49), knowledge scores (on a scale of 1–5) were significantly higher in those respondents who reported that their cat did not misbehave (cats do not misbehave: median = 4.00, range: 2–5, *n* = 288; cats misbehave: median = 3.67, range: 1–5, *n* = 257) (U = 31,109, *p* < 0.002). Female respondents had slightly higher knowledge scores (median = 4.00, range: 1–5) than male respondents (median = 3.67, range: 1.3–5) (H = 7.105, df = 1, *p* < 0.009); neither age of respondent nor duration of cat ownership were significantly correlated with knowledge score (*p* = 0.03 and *p* = 0.07, respectively). There was no significant difference between male and female respondents’ tendency to use positive punishment in response to perceived misbehavior (*p* = 0.693), or between households with varying numbers of cats (coded as 1, 2, 3 or more cats) (*p* = 0.295). No significant differences were seen in the knowledge score between households with varying numbers of cats (*p* = 0.044). For respondents who reported experiencing behavior problems with their cat, there were significant inverse relationships between respondents’ knowledge scores and the degree to which these behavior problems bothered them, for all potential behavior problems surveyed except housesoiling (where the inverse relationship was not statistically significant; *p* = 0.09) (Table 5).

For all cats, there was a positive correlation between the ratio of litterboxes/cats and the ratio of food stations/cats (r = 0.471, *p* < 0.001). The resources available per cat declined as the number of cats in the household increased: for litterboxes, mean number per cat declined significantly from 2.2 ± 0.5 in single-cat households, to 0.9 ± 0.4 in households of three or more cats (H = 390.567, df = 2, *p* < 0.0001). Similarly, the mean number of feeding stations per cat declined from 1.2 ± 0.5 in single-cat households to 0.5 ± 0.2 in households of three or more cats (H = 286.062, df = 2, *p* < 0.0001).

Significantly more behavior problems were reported for cats that were allowed outdoors (mean: 4.3 ± 3.0, *n* = 216) compared to indoor only cats (mean: 3.3 ± 2.2, *n* = 328) (U = 29,948, *p* < 0.003). There was no significant relationship between the number of hours the cat was left alone (with no human present) and the number of behavior problems reported (*p* = 0.464).

There was also no relationship (under the more conservative *α* = 0.01) between the number of dogs in the household (0, 1, 2 or more) and whether or not the owner reported that the cat misbehaved, based on a chi-square analysis (although the results approached significance, with more problems reported in households with 2 or more dogs; *p* = 0.017) (Table 6). There was no relationship between number of children ≤10 years of age in the household (0, 1, 2 or more) and whether or not the cat misbehaved (*p* = 0.144) (Table 6). Similarly, based on a Kruskal-Wallis test, there was no relationship between the number of cats in the household (1, 2, 3 or more) and the number of behavior problems reported in the focal cat (*p* = 0.168).

#### 3.2.3. Relationships between the Measures of the Human-Animal Bond and Characteristics of the Cat, Household, Care Practices, and Owner Attitudes

##### Knowledge, Use of Positive Punishment, and the Human-Animal Bond

When considering the number of potential behavior problems reported by owners, there were significant negative relationships seen between the three bond subscores and the number of behavior problems (interactions: *r* = −0.213; emotional bond: *r* = −0.186; affordability: *r* = −0.434; all *p* < 0.0001) (Table 4). Conversely, there was no significant difference between owners who stated that their cat misbehaved (*n* = 257) and those whose cats did not misbehave (*n* = 288) in the interactions subscore (*p* = 0.069) nor the emotional bond subscore (*p* = 0.567), but cat owners who stated their cats did misbehave had significantly lower perception of affordability than owners whose cats did not misbehave (U = 26,995, *p* < 0.0001). For all respondents, the three bond-related subscores were significantly and positively correlated with each other (Table 4).

Significant positive correlations were found between respondents’ bond with their cats (as reflected in the three bond subscores) and their knowledge score (interactions: *r* = 0.313; emotional bond: *r* = 0.388; affordability: *r* = 0.430; all *p* < 0.0001) (Table 4). For respondents who reported that their cat misbehaved (i.e., answered ‘yes’ when asked whether their cat ever misbehaved; *n* = 257), there were weak but significant negative relationships between the use of positive-punishment based approaches and bond subscores for interactions (*r* = −0.175, *p* < 0.006) and affordability (*r* = −0.224, *p* < 0.001), but not for emotional bond (*r* = −0.144, *p* = 0.021).

##### Children, Dogs, Multiple Cat Households, Owner Gender, and the Human-Animal Bond

When comparing owners’ bond with their cat in households with children (*n* = 219) vs. households without children (*n* = 326), emotional bond and affordability subscores were both significantly higher in households without children (emotional bond: H = 12.104, df = 1, *p* < 0.002; affordability: H = 12.407, df = 1, *p* < 0.0001). There was no difference in the interactions subscore for households with vs. without children (*p* = 0.217). There were no differences between households with dogs (*n* = 293) vs. without dogs (*n* = 251) on any of the bond subscores (interactions: *p* = 0.934, emotional bond: *p* = 0.105, affordability: *p* = 0.221). The emotional bond subscore was higher in homes with three or more cats (H = 18.323, df = 2, *p* < 0.0002); no significant differences were seen in the other bond-related measures and number of cats in the household (interactions: *p* = 0.623; affordability: *p* = 0.344). All three bond-related subscores tended to be higher in female respondents than males, but only the emotional bond subscores were significantly higher (emotional bond: H = 9.148, *p* < 0.003; affordability: *p* = 0.011; interactions: *p* = 0.055).

##### Cat Characteristics (Declaw Status, Outdoor Access, and Source) and the Human-Animal Bond

When the bond-related subscores for owners of cats that were not declawed (*n* = 417), already declawed when obtained (*n* = 61), or declawed by the respondent (*n* = 67), were compared, scores were significantly different between groups for all three bond-related subscores (interactions: H = 16.162, df = 2, *p* < 0.001; emotional bond: H = 26.472, df = 2, *p* < 0.0001; affordability: H = 35.111, df = 2, *p* < 0.0001). However, in examining the pairwise comparisons for each test, results were complex. There was no difference in any of the subscores between owners of cats who were not declawed, and owners who had declawed their cat (*p* > 0.11 for all). However, there were significantly lower scores for all three bond measures in owners of cats who were declawed prior to being obtained, compared to owners of cats who were not declawed (interactions: *p* < 0.002; emotional bond: *p* < 0.0001; affordability: *p* < 0.0001). For affordability only, scores for owners of cats who were declawed when obtained were significantly lower than scores for owners who declawed their cat (*p* < 0.008). Bond-related subscores were significantly higher for indoor-only cats than for cats allowed outdoors (interactions: H = 24.224, df = 1, *p* < 0.0001; emotional bond: H = 13.016, df = 1, *p* < 0.0001; affordability: H = 10.223, df = 1, *p* < 0.002). There were no differences seen in subscores on any of the three bond-related measures between owners of cats from different sources (e.g., shelter/rescue, friend/family, found as stray; see Table 1 for all sources surveyed) (*p* > 0.10 for all).

##### Owner Attitudes about Cats as Pets, Use of Positive Punishment, and the Human-Animal Bond

There were significant correlations, both positive and negative, between some owner attitudes and the occurrence of behavior problems, owner use of punishment, and the three bond measures (Table 7). For example, there were significant inverse relationships between agreement with statements like “Cats don’t like to play with their owners”, “Cats are naturally antisocial, so they don’t like living with other cats”, and “Cats do not care when their owners are gone” and all three bond-related measures; agreement with these statements was also positively correlated with the number of behavior problems reported (all *p* < 0.0001) (Table 7). There were significant positive correlations between agreement with the statement “Cats often misbehave (for example, by urinating outside the litterbox) to get back at their owners for doing something the cat did not like” and both the number of reported behavior problems (r = 0.263, *p* < 0.0001) and the tendency to use positive punishment (r = 0.205, *p* < 0.0001).

### 3.3. Predictors of Behavior Problems in Owned Cats (Multiple Linear Regression Analysis)

The multiple regression model predicting the number of reported behavior problems using owner practices and attitudes was significant (F10, 246 = 10.521, *p* < 0.0001), with an *R*^2^ of 0.3. Significant predictors of number of behavior problems in the model included owner knowledge score (B = −0.397; *p* < 0.04), pet-owner interaction subscore (B = −1.249; *p* < 0.0001) and the affordability subscore (B = −0.681; *p* < 0.0001). Complete results for the regression analysis, including model coefficients for each independent variable, are shown in Table 8.

## 4. Discussion

Taken as a whole, the results of this study supported the original hypothesis: owners with a more accurate understanding of cat behavior reported fewer behavior problems in their pets. Greater owner knowledge about cats was correlated with fewer reported behavior problems, less use of positive-punishment-based responses to misbehavior, and increased tolerance of potential behavior problems when these behaviors were reported (with the exception of housesoiling). The reason why housesoiling did not fit the pattern (i.e., higher knowledge associated with higher tolerance) cannot be determined from these correlational results. One possible explanation may be that housesoiling is often indicative of a medical problem, which may be difficult to resolve, particularly if persistent stressors within the cat’s environment are a contributing factor. Alternately, this discrepancy may be related to the fact that housesoiling was the behavior reported most bothersome by owners who experienced this with their cats, despite the fact that it was not the most commonly reported behavior problem. Housesoiling, when it occurs (and regardless of whether a behavioral or medical issue), may understandably be problematic for all cat owners, given its potential for damaging property, creating unpleasant odors, and adding to the cost (time and money) of caring for the cat. This is consistent with reports in the literature noting that housesoiling is a primary cause for relinquishment of cats to shelters [20,26].

In addition, these results supported the hypothesis that owners with stronger perceived bonds with their cats would report fewer behavior problems. There were significant inverse relationships between the number of reported behavior problems and all three human-animal bond subscores used in this study, and two of these bond measures (owner interactions and perception of affordability) were significant predictors of the number of reported behavior problems in the multiple regression analysis. This finding supports previous research that has suggested that behavior problems can cause damage to the human-animal bond [18]. Correspondingly, an owner’s tendency to use positive punishment, a risk factor for decreased welfare [21], was inversely related to measures of owners’ interactions with their pets, and positively related to how costly they felt it was to care for the cat (the more costly they perceived their cat’s care, the more likely they were to use positive punishment). Interestingly, the inverse relationship between use of punishment and the emotional bond between owner and cat did not reach statistical significance, perhaps highlighting the need for additional education of well-intentioned cat owners about the welfare risks to their cats of using positive punishment-based approaches.

Similarly, owner attitudes, particularly belief in common misconceptions about cat behavior, may contribute to suboptimal care of cats in the home. For example, respondents’ agreement with the statement, “Cats often misbehave (for example, by urinating outside the litterbox) to get back at their owners for doing something that the cat doesn’t like” was positively correlated with their tendency to use positive punishment. There were also direct relationships between agreement with statements like, “Cats don’t like to play with their owners” and “Cats do not care when their owners are gone” and the number of behavior problems reported, as well as inverse relationships between agreement with these types of statement and all three measures of the cat-owner bond: the more bonded an owner was to their cat, the less likely they were to agree with these statements. Given the importance of the bond to the care and welfare of the cat [4,8,9,13], it would suggest that correcting these types of misconceptions could be beneficial to standards of care for privately-owned cats.

An additional study goal was to identify gaps in current cat owner knowledge and pet care practices as a prelude to further research to demonstrate causal relationships and the creation of educational campaigns to correct misconceptions and improve owner knowledge. Many respondents in this study reported using positive punishment-based responses to misbehavior, including a small proportion who reported hitting or kicking their cat. A preponderance of studies of domestic dogs have linked the use of positive punishment to an increased risk of behavior problems such as fear-based aggression and compromised welfare (see Ziv [27] for a recent review). Although research is lacking on this relationship in cats, a number of authors recommend avoiding direct, physical, interactive punishment with cats, as it is likely to cause cats to become fearful and potentially defensive (e.g., references [28,29]). A relationship between increased use of positive punishment-based approaches and an increased number of behavior problems was seen in this study, but as this is correlational, it cannot be definitively stated that one caused the other, or the direction of causality if it exists. In addition, some owners in the present study (12.3%) had had their own cats declawed, and an additional 11.2% of cats had been declawed prior to being obtained by the present owner. This practice continues despite concerns about the welfare impact of declawing, and the link documented in a number of recent studies between declawing and behavior problems (e.g., reference [30]). Behavior problems were significantly more prevalent in declawed cats in the current study, supporting these findings. In addition, bond-related scores were significantly lower in owners of cats who had been declawed prior to adoption compared to owners of cats that were not declawed, but no significant differences were found in the measures of the owner-cat bond between owners who had declawed their cats and owners of cats that had not been declawed. As with the continued use of positive punishment by some owners, this finding may highlight the need for better education of well-meaning cat owners as to the risks of declawing to health and welfare of their cats. The reason for the apparent lower bond (representing a more negative view of the cat) in owners of cats that were declawed prior to adoption (vs. owners who had their cat declawed) is not clear. It may be, given the higher incidence of behavior problems in declawed cats seen in this study and others, pre-existing behavior problems interfered with the development of a strong bond between these cats and their new owners. Additional research is needed to answer this question. It is also important to note that the declawing may have been performed on some cats as a result of an existing behavior problem; the present cross-sectional study is unable to determine which sequence of events (i.e., declawing as cause, or result, of a behavior problem) is the correct one (and it may vary by cat).

Intercat aggression is a frequently reported behavior problem in privately-owned cats [6], and aggression between pets is a common reason for relinquishment of owned cats [19,26]; so unsurprisingly, there has historically been concern about increased social and territorial stress levels of cats living in multi-cat homes (e.g., references [4,13,31]). As noted earlier, environmental stressors can contribute to behavior problems in domestic cats [8] and stressors can include high densities of conspecifics and/or insufficient resources [4]. Yet, when housed together, unrelated cats often appear to avoid overlap and potential conflict by partitioning available resources (such as comfortable resting places or feeding stations) by frequenting different parts of the home, or by using resources at differing times of day [32]. In fact, studies of basal urinary cortisol [5] and fecal cortisol [33] found no differences between these measures in cats living in single-cat, vs. multi-cat, homes. Similarly, in the present study, no significant differences were found between the number of behavior problems reported for focal cats living in homes housing 1, 2, or 3 or more cats. As Ramos et al. [34] and others have noted, other environmental factors in the home, such as resource availability, may be a more important influence on cat behavior than simply the number of cats present. Thus, given the reported prevalence of intercat aggression (particularly among unrelated cats forced to live in close proximity), it is concerning that the number of litterboxes and separate feeding stations available per cat declined as the number of cats in the household increased. This effect was particularly marked once the number of cats in the household reached 3 or more.

Equally concerning is the number of cats that were offered minimal environmental enrichment. Nearly one-third of cats were left alone for 5–8 h per day (presumably while their owners were at work), with more than 13% left alone for longer periods. Only about half of the respondents reported that they play with their cats every day, and (as training can be considered a form of enrichment) only about one-third reported training their cat in any way. Additionally, 60% of cats in this study were kept indoors only (i.e., without access to the sensory stimulation and changing environment provided by spending time outdoors). Although the majority of respondents reported providing some forms of static environmental enrichment (such as toys, and private sleeping areas) for their cats, taken together (i.e., time alone, indoor only, and provision of environmental enrichment) these numbers suggest that a significant proportion of cats may not be getting the amount of active/interactive enrichment (such as positive interactions with a familiar human) generally recommended by cat researchers and experts. If true, this may partially explain the results of the present study, in which up to 60% of owners reported experiencing one or more of the surveyed behavior problems in their cats (Table 3). This is a similar proportion to that reported recently by Strickler and Shull [6], in which 61% of the owners reported that their cat engaged in one or more of the six behavior problems surveyed in that study, but lower than another recent survey of North American cat owners [33], in which 97.8% of owners reported experiencing at least one behavior problem in their cats. In the present study, over 50% of respondents reported cat vomiting, considered a sign of stress in cats [35,36]. Many owners appear to assume that some vomiting is normal for cats, when it may be an indication of an undiagnosed gastrointestinal issue; this may be another area for continuing education of cat owners. The frequency of this behavior, however, was not investigated in the present study, so additional research pertaining to the frequency of this behavior and links to environmental stressors on cats living in private homes is warranted.

In the present study, behavior problems were more commonly reported in cats allowed outdoors than for fully indoor cats, as has been reported elsewhere [16]; and measures of the owner-cat bond were higher for indoor-only cats than for cats allowed outdoors. Based on this correlational analysis, however, it is not possible to say whether or not outdoor access caused behavior problems; it may be that cats exhibiting behavior problems were more likely to be ‘evicted’ from the home to some degree by frustrated owners. This latter explanation would fit with the tendency for bond measures to be lower for cats allowed outdoors, and lower for cats exhibiting behavior problems, as seen in this study. Allowing pet cats access to the outdoors has become controversial, with some experts noting that access to the outdoors (e.g., supervised and/or safe spaces) can be beneficial [13,17]. Outdoors may also provide a way for cats to distance themselves from socially incompatible feline housemates [4], and fully indoor life is sometimes associated with conditions and diseases such as obesity, hyperthyroidism, and behavioral problems [37]. However, there are also significant risks associated with access to the outdoors [37], to both cats and wildlife. The American Veterinary Medical Association (AVMA) currently encourages veterinarians to educate their clients and the public about these risks and that “keeping owned cats confined, such as housing them in an enriched indoor environment, in an outdoor enclosure, or exercising leash-acclimated cats, can minimize the risks to the cat, wildlife, humans, and the environment” [38].

Collectively, the results of this cross-sectional analysis suggest the need for better education of cat owners. Despite the efforts of many animal sheltering and welfare organizations, veterinarians and behaviorists, it can be challenging for pet owners to obtain consistent, accurate, evidence-based information about responding to pet behavioral needs and problems, as has been discussed elsewhere [33,39,40]. Veterinarians seeking up-to-date information to share with their clients can refer to documents such as the 2013 AAFP and ISFM Feline Environmental Needs Guidelines [17] and the 2015 AAHA Canine and Feline Behavior Management Guidelines [41]. Changing human behavior is, of course, a challenging and complex endeavor (see reference [42] for an example of this issue relative to domestic cats]. While a summary of that literature is beyond the scope of this paper, it may be helpful when designing educational campaigns to refer to the principles of adult learning, such as discussed in the work of Vella [43] and Zull [44]; for an example of this approach with adopters in an animal sheltering context, see Troughton [45].

Providing links to helpful websites where information is easy to find, well-organized, and attractively presented may appeal to many cat owners; such as the International Cat Care site (https://icatcare.org/); or the impressive selection of short, informative videos on sites like Cat Protection’s YouTube channel (https://www.youtube.com/user/catsprotectionuk). The results of the present study suggest that specific topics to be addressed include: correcting common negative misconceptions about cats and cat behavior; normal cat behavior and enrichment needs (particularly indoor-only cats) (see reference [4,13,38,46]); the risk of behavior problems when cats’ needs are not met; welfare risks associated with declawing; the benefits of owning a cat (to offset increased perception of costs in some owners); medical issues that may trigger some types of behavior (such as vomiting and housesoiling); and the importance of sufficient resources to minimize conflict between cats and associated social stressors (particularly in homes with three or more cats, where providing sufficient resources may require more time and conscientious effort).

This study was subject to the following limitations: the data collected represents a snapshot of attitudes and practices of cat owners in the United States at one point in time (August 2019). As the survey was administered online, it was not available to U.S. cat owners who do not have access to the internet, and was limited to respondents who were comfortable answering questions in English. Information on cat behavior problems, owner practices, etc., was self-reported, and thus was potentially subject to differences in interpretation, especially in terms of what constitutes “misbehaving”. Additionally, some owners may have been unwilling to admit using certain practices in response to their cats’ misbehavior. Nonetheless, the diversity of respondents and the quality of survey response data obtained though the MTurk platform (see reference [23]), along with the agreement between many of the current findings and results reported elsewhere, collectively support that these results are representative of a large proportion of U.S. cat owners.

In summary, this was a descriptive, correlational study, designed to identify current attitudes towards pet cats, prevailing cat care practices in private homes, and to investigate relationships between these variables and the behavior (as an index of welfare) of privately-owned cats. As noted elsewhere in the paper, these results do not allow establishment of cause and effect relationships between variables; rather, the relationships and patterns identified in this study serve to inform future research and educational outreach aimed at improving the lives of companion cats and their owners.

## 5. Conclusions

The primary hypothesis was supported: greater owner knowledge about cats was related to fewer reported behavior problems, less use of positive-punishment-based responses to misbehavior, and increased tolerance of potential behavior problems when present. Owners with stronger bonds with their cats reported fewer behavior problems. Conversely, owners’ agreement with certain misconceptions about cats, and perception of high costs of care, were correlated with use of positive punishment in response to misbehavior. As reported elsewhere, declawed cats exhibited more behavior problems than cats who retained their claws. Based on the survey results, many cats living in private homes may be receiving only minimal environmental enrichment, particularly in the case of interactive (vs. static) enrichment. Collectively, this needs assessment supports the premise that better education of cat owners would benefit the welfare of cats living in private homes. Recommended topics include: understanding normal cat behavior and correcting misconceptions; enrichment needs (particularly of indoor-only cats) and the risk of behavior problems when cats’ needs are not met; welfare risks associated with declawing; and the importance of sufficient resources to minimize social and territorial conflict.

## Figures and Tables

**Table 1:** Demographic data on U.S. cat owners (**a**) and their cats (**b**) (*n* = 547 respondents). For ease of interpretation, results are presented here to two significant figures.

(a) Humans:				
Age (years):		Gender:		
Mean (SD):	38 (11.5)	Percentages:	Male: 39%	
Median:	36		Female: 60%	
Range:	18–74		Non-binary/other: 0.6%	
Household composition:				
	Adults (≥18 years):	Children (≤10 years):	Cats in home:	Dogs in home:
Mean (SD):	2.1 (0.8)	1.7 (1.0)	1.8 (1.4)	1.8 (0.9)
Range:	1–5	1–6	1–11	1–6
Percentages:	1: 18%	0: 60%	Single cat: 58%	No dogs: 46%
	2: 62%	1: 20%	2 cats: 26%	1 dog: 35%
	3: 14%	2: 14%	3 or more: 16%	2 dogs: 14%
	4 or more: 6.4%	3 or more: 6.2%		3 or more: 4.4%
		Human-animal bond subscores *:		
Knowledge subscore *:		Pet-owner interactions:	Emotional closeness:	Affordability:
Median:	3.7	4.8	4.7	4.3
Range:	1–5	2–5	1–5	1–5
**(b) Cats:**				
Current age (years):		Age (years) when obtained:		
Mean (SD):	6.9 (4.6)	Percentages:	<1: 72%	
Median:	5.0 (mode = 2)		1: 13%	
Range:	1–18		2: 7.5%	
			3: 3.3%	
			4: 1.3%	
			5 or more: 3.0%	
Sex:				
Percentages:		Male (neutered): 40% Male (intact): 8.2% Female (spayed): 45% Female (intact): 7.0%		
Is the cat declawed?				
Percentages:		No: 77%		
		Yes, he/she was when I got him/her: 11%		
		Yes, I had him/her declawed: 12%		
Source of cat:		Duration of ownership (years):		
Percentages:	Shelter/rescue: 42%	Percentages:	<1: 6.4%	
	Friend/family: 27%		1–2: 27%	
	Found as stray: 19%		>2–5: 29%	
	Breeder: 2.4%		>5–10: 20%	
	Pet store: 5.8%		>10: 18%	
	Other: 4.2%			

* Higher scores represent better knowledge (knowledge subscore) and relationship with the cat (bond subscores). Calculation of the subscores is explained in the text; bond subscores are adapted from Howell et al. (2017) [9].

**Table 2:** Resources provided to companion cats living in homes (*n* = 547 respondents).

Environmental Resource	Percentage of Owners Who Provided This Resource	
Quiet, private hiding place(s)	92.1%	
Toy(s) for independent play (such as ping pong balls, catnip-stuffed toys, puzzle toys, etc.)	81.3%	
Window seat/perch with view of outside	79.0%	
Comfortable cat bed(s)	75.9%	
Scratching post indoors	68.7%	
Climbing perch and/or cat furniture (cat trees, etc.)	51.7%	
Training (to do tricks, use the toilet, play fetch, etc.)	30.7%	
Calming pheromone product (e.g., Feliway)	6.5%	
Resource provided per cat	Number provided per cat (mean ± SD; range)	
Litterboxes	1.7 ± 0.7; range: 0.1–4	
Feeding stations	1.0 ± 0.5; range: 0.1–3	
Resource provided per time interval	Percentage by Frequency or Duration	
Interactive play (with owner; frequency)	Daily or more:	47.7%
	Once every few days:	32.5%
	Once a week:	11.3%
	Once a month:	4.6%
	Never:	3.8%
Access to outdoors (h)	0 (indoor only):	60.0%
	1–2 h:	20.3%
	3–4 h:	7.7%
	5–6 h:	2.6%
	>6 (but not all the time):	5.3%
	24 (outdoors only):	4.2%
Time spent alone (without humans; h)	None:	11.2%
	1–2:	20.7%
	3–4:	24.0%
	5–8:	30.7%
	9–12:	9.3%
	>12:	4.2%

**Table 3:** Prevalence of surveyed potential feline behavior problems, and (for owners encountering these issues) the degree to which the behavior bothered the cat owner.

Question: “Please indicate if your cat exhibits any of the following behaviors and how bothered by this behavior you or someone else in your household is:”	Reporting Behavior Not Seen	Reporting Behavior	Percentage Responses among Owners Reporting the Behavior in Their Cats:			
			Not Bothered at All	Bothered a Little Bit	Bothered a Fair Amount	Bothered a Great Deal
Aggression towards familiar people (such as you or other members of the family)	75.7%	24.3%	37.6	35.3	21.1	6.0
Aggression towards unfamiliar people such as visitors to the home	74.4%	25.6%	42.9	35.7	17.1	4.3
Aggression towards other non-human animals in (or around) the home	56.9%	43.1%	51.7	30.9	12.7	4.7
Anxiety or fear (e.g., fear of strangers, travel, of carrier, etc.)	40.6%	59.4%	48.3	29.5	17.2	4.9
Excessive vocalization (including nighttime vocalization)	54.7%	45.3%	45.6	33.5	14.1	6.9
Destructive behavior (e.g, scratching furniture)	50.3%	49.7%	37.9	36.4	18.0	7.7
Obsessive or repetitive behaviors such as shadow (or light) chasing, pacing, chasing his/her own tail	60.0%	40.0%	61.6	19.6	14.6	4.1
Housesoiling (urination or defecation outside litter box, etc.)	73.5%	26.5%	37.9	29.7	24.1	8.3
Vomiting/throwing up (food, grass, hairballs, other)	45.9%	54.1%	51.0	31.1	13.5	4.4

**Table 4:** Correlation matrix for knowledge score (higher score reflects more accurate understanding of cat behavior), number of behavior problems reported by owner, use of positive punishment-based responses to perceived misbehavior (higher score indicates more punishment used) and three bond-related measures: interactions (between owner and cat), emotional bond, and affordability. Bond subscores were adapted from Howell et al. (2017) [9]. For the three bond-related subscores, a higher score reflects a more positive relationship with the cat (e.g., a lower ‘affordability’ score meant that the owner was more bothered by the costs associated with caring for their cat). **Bold font** indicates that the correlation is significant from 0 at *p* < 0.0001; *italic font* indicates that the correlation is significant but at a different level; see footnotes for these *p* values.

Variables	Knowledge	Number of Behavior Problems	Use of Positive Punishment	Interactions Subscore	Emotional Bond Subscore	Affordability Subscore
Knowledge	**1**					
Number of behavior problems	**−0.325**	**1**				
Use of positive punishment	**−0.183**	**0.407**	**1**			
Interactions subscore	**0.313**	**−0.213**	−*0.175* *^,1^	**1**		
Emotional bond subscore	**0.388**	**−0.186**	−0.144 *	**0.426**	**1**	
Affordability subscore	**0.430**	**−0.434**	−*0.224* *^,2^	**0.302**	**0.465**	**1**

* for these correlations, only respondents who had reported that their cats did misbehave were included in the calculations. ^1^ *p* < 0.006, ^2^ *p* < 0.001.

**Table 5:** Correlations (Spearman’s) between owner knowledge (higher score reflects more accurate understanding of cat behavior) and tolerance for undesirable behaviors exhibited by their cat (higher score indicates that the owner is more bothered by this behavior). Owners who reported that their cat did not exhibit the behavior were removed from this analysis; response options ranged from 2 (“My cat does exhibit this behavior, but I’m not bothered by it at all”) to 5 (“My cat does exhibit this behavior, and it bothers me a great deal”). **Bold font** indicates that the correlation is significant from 0 at *p* < 0.0001; *italic font* indicates that the correlation is significant but at a different level; see footnotes for these *p* values.

Potential Behavior Problem	Correlation
Aggression towards familiar people (such as you or other members of the family)	**−0.363**
Aggression towards unfamiliar people (such as visitors to the home)	**−0.349**
Aggression towards other non-human animals in (or around) the home	**−0.259**
Anxiety or fear (of strangers, travel, etc.)	*−0.160* ^1^
Excessive vocalization (inc. during nighttime)	**−0.298**
Destructive behavior (such as scratching furniture)	*−0.184* ^2^
Obsessive/repetitive behaviors (such as shadow or light chasing, chasing his/her own tail, etc.)	**−0.324**
Housesoiling (urination or defecation outside the litterbox)	−0.142
Vomiting (food, grass, hairballs, etc.)	*−0.182* ^2^

^1^*p* < 0.005, ^2^ *p* < 0.003.

**Table 6:** Contingency tables for household demographics and owner-reported misbehavior (yes/no).

(a) “Does Your Cat Misbehave?” and “Number of Dogs in Household” (χ = 8.112, df = 2, *p* = 0.017)			
	Yes (Misbehaves)	No (Does Not Misbehave)	Totals:
No dogs	116	136	252
1 dog	81	111	192
2 or more dogs	60	41	101
Totals:	257	288	545
(b) “Does Your Cat Misbehave?” and “Number of Children in Household” (χ = 3.872, df = 2, *p* = 0.144)			
	Yes (Misbehaves)	No (Does Not Misbehave)	Totals:
No children	152	174	326
1 child	58	48	106
2 or more children	47	66	113
Totals:	257	288	545

**Table 7:** Correlations between owner attitudes (higher score reflects stronger agreement with the statement), number of behavior problems reported by owner, use of positive punishment-based responses to perceived misbehavior (higher score indicates more punishment used) and three bond-related measures: interactions (between owner and cat), emotional bond, and affordability (higher score reflects a more positive relationship with the cat, e.g., a lower ‘affordability’ score meant that the owner was more bothered by the costs associated with caring for their cat). Coefficients shown in **bold font** indicates that the correlation is significant from 0 at *p* < 0.0001; *italic font* indicates that the correlation is significant but at a different level; see footnotes for these *p* values.

Variables	Number of Behavior Problems	Use of Positive Punishment	Interactions Subscore	Emotional Bond Subscore	Affordability Subscore
Cats don’t like to play with their owners	**0.242**	0.105	**−0.378**	**−0.464**	**−0.392**
Cats are naturally aloof and independent	0.107	*0.143* ^1^	−0.022	−0.109	−0.080
Cats like to live with another cat in the household	0.054	−0.042	0.031	*0.148* ^1^	0.001
The only reason cats spend time with humans is because humans feed them	**0.281**	*0.133* ^2^	**−0.322**	**−0.437**	**−0.413**
Cats are low-maintenance pets	*−0.131* ^2^	−0.091	0.040	0.076	**0.128**
Cats should be kept indoors all the time	0.052	0.005	0.092	*0.134* ^2^	0.032
Cats can’t be trained to do tricks	**0.185**	0.093	**−0.262**	**−0.279**	**−0.277**
Cats do not care when their owners are gone	**0.225**	0.083	**−0.364**	**−0.425**	**−0.336**
Cats need to spend time outside to be happy	*0.135* ^2^	0.082	**−0.170**	**−0.167**	**−0.192**
Cats can be just as strongly bonded to their owners as dogs can be	*−0.163* ^2^	−0.023	**0.314**	**0.555**	**0.396**
Cats often misbehave to get back at their owners for doing something the cat did not like	**0.263**	**0.205**	−0.069	−0.049	**−0.222**
Cats are naturally antisocial, so they don’t like living with other cats	**0.311**	*0.126* ^3^	**−0.316**	**−0.345**	**−0.332**

^1^*p* < 0.002, ^2^ *p* < 0.003, ^3^ *p* < 0.004.

**Table 8:** Results of the multiple linear regression model predicting the number of reported behavior problems in domestic cats as a function of owner and household characteristics.

ANOVA						
Model	Sum of Squares	df	Mean Squares	F	Sig.	
Regression Residual Total	417.468 976.151 1393.619	10 246 256	41.747 3.968	10.521	0.000	
**Coefficients** (Dependent Variable: Number of Reported Behavior Problems). Significant Predictors Are Shown in **Bold**						
Variable			Coefficient (B)	Std. Error	*t*	Sig.
(Constant)			12.973	1.582	8.202	0.000
Total number of cats in the home			0.043	0.122	0.350	0.727
Number of dogs in the home			−0.228	0.122	−1.868	0.063
Number of children in the home			0.073	0.131	0.561	0.575
**Knowledge subscore** (higher score reflects better knowledge)			**−0.397**	**0.190**	**−2.087**	**0.038**
Litterbox ratio (number of boxes/number of cats in home)			0.048	0.278	0.172	0.863
Feeding stations ratio (number of separate food bowls/number of cats)			−0.292	0.310	−0.939	0.349
Use of positive punishment (higher score reflects more punishment used)			0.356	0.289	1.229	0.220
**Pet owner interaction subscore** *			**−1.249**	**0.243**	**−5.146**	**0.000**
Emotional closeness subscore *			0.252	0.204	1.231	0.220
**Affordability subscore** *			**−0.681**	**0.162**	**−4.216**	**0.000**

*R*^2^ = 0.300. * higher bond subscores indicate a more positive relationship with the cat; bond subscores were adapted from Howell et al. (2017) [9].

## Supplementary Materials

The following are available online at https://www.mdpi.com/2076-2615/9/11/978/s1, Sixty-seven-item cross-sectional survey use in the study.
