# Supplementary material for: Owners’ Attitudes, Knowledge, and Care Practices: Exploring the Implications for Domestic Cat Behavior and Welfare in the Home
Source: Animals (Basel). 2019 Nov 15;9(11):978. doi: 10.3390/ani9110978 (PMC6912669; doi:10.3390/ani9110978)
Supplement: Supplementary file 1 [file animals-09-00978-s001.pdf]

animals

Supplementary Materials: Owners' Attitudes, Knowledge, and Care Practices: Exploring the Implications for Domestic Cat Behavior and Welfare in the Home

Emma K. Grigg [1,*] and Lori R. Kogan [2]

[1] Department of Population Health and Reproduction, School of Veterinary Medicine, University of California, Davis, CA 95616, USA
[2] Clinical Sciences Department, Colorado State University, Fort Collins, CO 80523, USA; lori.kogan@ColoState.edu
* Correspondence: ekgrigg@ucdavis.edu

Cat Behavior Survey

Cat owners-

Your experiences and perceptions are wanted. We are researchers from Colorado State University and University of Davis California and we want to better understand people's knowledge about cats as well as their cat's behaviors.

We are specifically looking for cat owners who are the primary caretaker of a cat between the ages of 1–18 years of age and have owned this cat for at least 6 months.

If you meet these qualifications, we would like to ask you to please consider taking the following short anonymous survey.

Fine Print:

What is involved?

You will be asked to complete a series of questions as honestly as possible and there are no right or wrong answers. The questionnaire should take no more than 10 min to complete. Participation is entirely voluntary. You may quit at any time.

Are there any benefits or risks in my taking part?

There are no direct risks or benefits to completing the survey. The survey is voluntary and anonymous and you may stop the survey at any time by closing the window. Data from the survey will be used only for research and will hopefully be published in a journal.

Will my participation be confidential?

Yes, all participation will be confidential. The data will be anonymous and will contain no information that could lead to the identity of individuals. Anonymous data will be kept on a password protected computer.

What happens if I change my mind?

If you feel you do not wish to continue with the questionnaire, you can close the browser window.

Where can I get more information?

If you have questions about this research please contact Dr. Lori Kogan (Lori.Kogan@ColoState.EDU). Any questions about participant rights related to this survey can be directed to CSU IRB (ricro_irb@mail.colostate.edu) or 970 491-1655. Questions about the survey can be directed to Lori Kogan, PhD at lori.kogan@colostate.edu.

Consent

I have read and understood the information given above. In consenting, I agree to take part in this research project and agree for my data to be used for the purpose of this study. I understand that my participation is voluntary and I may withdraw at any time.

Please Tell Us a Bit about Yourself

[1] Have you been the primary caretaker of cat between the ages of 1–18 years old for at least six months?

○ Yes

○ No

[2] Are you 18 years old or older?

○ Yes

○ No

[3] Please tell us how old you are: ______________________________

[4] Please tell us your gender:

○ Male

○ Female

○ Non-binary

○ Prefer to not say

[5] How many cats live in your home (total, including the one you are answering survey questions about)?

(options: 0,1,2,3,4, etc., to 10 and more than 10)

[6] How many dogs live in your home?

(options: 0,1,2,3,4, more than 4)

[7] How many adults-18 years or older- (total, including yourself) live in your home?

(options: 0,1,2,3,4, more than 4)

[8] How many children (10 years of age or younger) live in your home?

(options: 0,1,2,3,4, more than 4)

Please indicate your agreement level with the following statements about **cats as pets in general** (strongly disagree to strongly agree):

	**Strongly Disagree**	**Somewhat Disagree**	**Neither Agree Nor Disagree**	**Somewhat Agree**	**Strongly Agree**
[9] Cats don't like to play with their owners	○	○	○	○	○
[10] Cats are naturally aloof and independent	○	○	○	○	○
[11] Cats like to live with another cat in the household	○	○	○	○	○

[12] The only reason cats spend time with humans is because humans feed them	○	○	○	○	○
[13] Cats are low-maintenance pets	○	○	○	○	○
[14] Cats should be kept indoors all the time	○	○	○	○	○
[15] Cats can't be trained to do tricks	○	○	○	○	○
[16] Cats do not care when their owners are gone	○	○	○	○	○
[17] Cats need to spend time outside to be happy	○	○	○	○	○
[18] Cats can be just as strongly bonded to their owners as dogs can be	○		○	○	○
[19] Cats often misbehave (for example, by urinating outside the litterbox) to get back at their owners for doing something the cat did not like	○	○	○	○	○
[20] Cats are naturally antisocial, so they don't like living with other cats	○	○	○	○	○

Now, please tell us a bit about **<u>your cat</u>**. If you have more than one cat, choose the cat whose first name starts with the letter closest to the first letter of the alphabet, and answer the following questions only about this cat.

[21] How old is your cat (in years)?

(options: 6 months-1 year, then 1, 2, etc–through 18)

[22] What sex is your cat?

○ Male, intact

○ Male, neutered

○ Female, intact

○ Female, spayed

○ Don't know

[23] Where did you get your cat?

○ Shelter/rescue

○ Friend/family

○ Found stray

○ Breeder

○ Pet store

○ Other (please specify): ____

[24] How long have you owned your cat?

○ Less than 1 year

○ 1–2 years

○ >2 years–5 years

○ >5 years–10 years

○ Longer than 10 years

[25] Is your cat declawed?

○ Yes, he/she was when I got him/her

○ Yes, I had him/her declawed

○ No

○ I don't know

[26] How old was your cat when you got him/her?

(options: less than 1 year, 1,2, etc–through 18)

[27] On average, how much time (in hours) does this cat spend outdoors on any given day?

○ 0 h (indoor only cat)

○ Approximately 1–2 h

○ Approximately 3–4 h

○ Approximately 5–6 h

○ Over 6 h but not all the time

○ 24 h (outdoor only cat)

[28] On average, how much time does your cat spend alone (with no humans present) on an average day?

○ None

○ Approximately 1–2 h

○ Approximately 3–4 h

○ Approximately 5–8 h

○ Approximately 9–12 h

○ More than 12 h

[29] Is your cat currently on any medication?

○ Yes

○ No

[30] Please select for what reason your cat is taking medication:

○ Behavioral (e.g., aggression, anxiety)

○ Physical ailments (e.g., kidney, skin conditions)

○ Both behavioral and physical issues

For the following questions, tell us about *your cat's* behavior (answer the following questions in reference to the last 30 days of living with this cat):

Please indicate if your cat exhibits any of the following behaviors and how bothered by this behavior you or someone else in your household is:

	**NA (cat Does Not Exhibit This Behavior)**	**My Cat Does Exhibit This Behavior-But Not Bothered at All**	**My Cat Does Exhibit This Behavior-and Bothered a Little Bit**	**My Cat Does Exhibit This Behavior-and Bothered a Fair Amount**	**My Cat Does Exhibit This Behavior-and Bothered a Great Deal**
[31] Aggression towards familiar people (such as you or other members of the family)	○	○	○	○	○
[32] Aggression towards unfamiliar people such as visitors to the home	○	○	○	○	○
[34] Aggression towards other non-human animals in (or around) the home	○	○	○	○	○
[33] Anxiety or fear (e.g., fear of strangers, travel, of carrier, etc.)	○	○	○	○	○
[35] Excessive vocalization (including nighttime vocalization)	○	○	○	○	○

[36] Destructive behavior (e.g, scratching furniture)	○	○	○	○	○
[37] Obsessive or repetitive behaviors such as shadow (or light) chasing, pacing, chasing his/her own tail	○	○	○	○	○
[38] Housesoiling (urination or defecation outside litter box, etc.)	○	○	○	○	○
[39] Vomiting/throwing up (food, grass, hairballs, other)	○	○	○	○	○

[40] Have you ever considered relinquishing this cat because of his/her behavior?

○ Yes

○ No

[41] Has your cat's veterinarian ever (i.e., during the whole time that you have owned this cat) recommended that this cat be seen by a veterinary behaviorist or other animal behaviorist for a behavior problem (regardless of whether or not you followed this recommendation)?

○ No

○ Yes (please explain): ____

Next, Please Tell Us a Bit about *Your Cat's* Environment.

[42] Which of the following (if any) does your cat have access to? (select all that apply):

☐ Scratching post indoors

☐ Quiet, private hiding places

☐ Comfortable cat bed(s)

☐ Climbing perch and/or cat furniture (cat trees, etc)

☐ Window seat/perch with view of outside

☐ Toy for independent play (such as ping pong balls, catnip-stuffed toys, puzzle toys, etc.)

[43] Do you currently use Feliway or a similar calming pheromone product? (plug-in, collar, spray…)

○ Yes

○ No

○ Don't know

[44] How many litter boxes do you have in your house?

(options: 0,1,2,3,4, more than 4)

[45] What kind of litter do you currently use?

○ Clay clumping

○ Clay non-clumping

○ Litter pearls

○ Pine pellets

○ Corn-based

○ Multiple types

○ I don't know

○ Other (please specify) ____

[46] On average, how often do you scoop the litter box(es)?

○ More than once a day

○ Once a day

○ Once every other day

○ 2–3 times week

○ Once a week

○ Less than once week

[47] How many feeding stations (food bowls in different locations) does your cat have access to?

○ 1

○ 2

○ 3

○ More than 3

[48] Have you ever trained your cat to do tricks, use the toilet, play fetch, etc.?

- ◯ Yes
- ◯ No

[49] Does your cat ever misbehave (e.g., destructive scratching or inappropriate elimination)?

- ◯ Yes
- ◯ No

When your cat misbehaves, how often do you respond in the following ways?

	**Never**	**Seldom**	**Sometimes**	**Often**
[50] Yell	◯	◯	◯	◯
[51] Spray with water	◯	◯	◯	◯
[52] Hit/kick	◯	◯	◯	◯
[53] Make a loud noise (such as hand clapping)	◯	◯	◯	◯
[54] Ignore it	◯	◯	◯	◯
[55] Redirect my cat to a more acceptable behavior	◯	◯	◯	◯

[56] How often do you play games with your cat (such as with a cat 'fishing pole', or playing fetch, or chasing a laser pointer)?

- ◯ Daily or more
- ◯ Once every few days
- ◯ Once a week
- ◯ Once a month
- ◯ Never

The following questions ask you a bit more about your relationship with your cat:

	**At Least Once a Day**	**Once Every Few Days**	**Once a Week**	**Once a Month**	**Never**

[57] How often do you talk to your cat?	◯	◯	◯	◯	◯
[58] How often do you feel that looking after your cat is a chore?	◯	◯	◯	◯	◯
[59] How often do you have your cat with you while relaxing, e.g., watching TV?	◯	◯	◯	◯	◯
[60] How often do you pet your cat?	◯	◯	◯	◯	◯

The following questions ask about your relationship with your cat:

	**Strongly Disagree**	**Somewhat Disagree**	**Neither Agree nor Disagree**	**Somewhat Agree**	**Strongly Agree**
[61] There are major aspects of owing a cat I don't like	◯	◯	◯	◯	◯
[62] My cat helps me get through tough times	◯	◯	◯	◯	◯
[63] My cat is a member of my family	◯	◯	◯	◯	◯
[64] My cat makes too much mess	◯	◯	◯	◯	◯
[65] My cat provides me with constant companionship	◯	◯	◯	◯	◯
[66] My cat costs too much money	◯	◯	◯	◯	◯

[67] Additional comments about your cat or the relationship you have with your cat:

Thank you for completing the survey.
